# Ovarian dysfunction with moderate-dose intravenous cyclophosphamide (modified NIH regimen) and mycophenolate mofetil in young adults with severe lupus: a prospective cohort study

**DOI:** 10.1186/s13075-020-02292-y

**Published:** 2020-08-14

**Authors:** Shefali Khanna Sharma, Siddharth Jain, Pooja Bahl, Pragna Potturi, Manish Rathi, Shankar Naidu, Naresh Sachdeva, Varun Dhir, Sanjay Jain

**Affiliations:** 1 Clinical Immunology and Rheumatology Division, Department of Internal Medicine, Postgraduate Institute of Medical Education and Research, Chandigarh, India; 2 Department of Obstetrics and Gynaecology, Postgraduate Institute of Medical Education and Research, Chandigarh, India; 3 Department of Nephrology, Postgraduate Institute of Medical Education and Research, Chandigarh, India; 4 Department of Endocrinology, Postgraduate Institute of Medical Education and Research, Chandigarh, India

**Keywords:** Cyclophosphamide, Gonadotoxicity, Systemic lupus erythematosus, Mycophenolate mofetil, Ovarian dysfunction

## Abstract

**Background:**

Ovarian toxicity is a dreaded complication of cyclophosphamide (CYC). With the use of lower cumulative doses of intravenous CYC (modified NIH regimens) and availability of better markers of ovarian toxicity, the incidence of ovarian dysfunction needs reassessment. Lupus disease activity, by itself, is also believed to affect ovarian function negatively.

**Methods:**

This single-centre prospective cohort study recruited 50 female patients of severe lupus aged 18–40 years. Twenty-five patients each received induction with either monthly intravenous CYC (0.5–0.75 g/m^2^) for 6–9 months or daily oral mycophenolate mofetil (MMF). Details of menstrual irregularities; serum levels of FSH, LH, estradiol, AMH, and inhibin B; and sonographic assessment of ovarian volume and antral follicular count were done at baseline and 6 months after treatment. Amenorrhoeic patients were re-evaluated at 1 year.

**Results:**

Mean (SD) age of subjects in the CYC and MMF groups was 31.4 (6.3) and 28.4 (4.4) years, respectively. Mean (SD) SLEDAI at the initiation of therapy was 7.2 (2.5) in the CYC group and 5.8 (3.4) in the MMF group. The mean cumulative dose of CYC used was 4.6 (1.8) g. Three patients in the CYC group (versus none in MMF) had amenorrhoea at 6 months—two of these regained menses within 6 months, while only one (4%) developed sustained amenorrhoea (lasting more than 12 months) at 41 years of age, likely menopause. Serum FSH levels increased (*p* = 0.03), while AMH (*p* = 0.002) and inhibin B (*p* < 0.001) levels decreased significantly with 6 months of CYC therapy. Ovarian volume also reduced significantly (*p* = 0.005) with 6 months of CYC therapy, while antral follicular count reduced numerically (*p* = 0.32). Levels of AMH, inhibin-B, estradiol, ovarian volume, and antral follicular count after 6 months therapy were significantly lesser in the CYC group compared to the MMF group, despite being similar before the start of therapy.

**Conclusions:**

Ovarian dysfunction with monthly intravenous CYC (modified NIH regimen) was predominantly subclinical, with a negative effect on ovarian reserve. No premature ovarian failure was noted at 1 year. No ovarian dysfunction occurred in the MMF group, despite having patients with severe background lupus. Use of intravenous CYC for induction may thus not be restricted in young lupus females with incomplete families for fear of gonadotoxicity, especially in life- or organ-threatening situations, where the benefits outweigh this subclinical risk.

## Background

Systemic lupus erythematosus (SLE) is a prototypic autoimmune rheumatic disorder with a significant mortality and morbidity [1]. Cyclophosphamide (CYC) and mycophenolate mofetil (MMF) are potent first-line immunosuppressive agents used in the management of severe life- or organ-threatening manifestations, including lupus nephritis, myocarditis, neuropsychiatric lupus, lupus enteritis, diffuse alveolar haemorrhage, and secondary vasculitis [2]. Use of CYC, an alkylating agent, is associated with the risk of cytopenias, infections, malignancy, and gonadal toxicity [3]. A recently published study from our centre showed similar response rates of low-dose intravenous CYC and MMF in lupus nephritis; however, MMF was found to be more expensive and had more gastrointestinal side effects [4]. Besides, there is still limited evidence of the efficacy of MMF in certain severe manifestations of SLE like diffuse alveolar haemorrhage, crescentic lupus nephritis with renal failure requiring dialysis, and severe neuro-lupus, where CYC is still the favoured first-line treatment.

Since SLE predominantly affects young females belonging to the reproductive age group, the risk of premature ovarian failure associated with CYC hinders its use in these patients. The incidence of ovarian failure with CYC was reported to be between 11 and 59% as per a review in 2004 [5]. However, majority of these studies belonged to the pre-2000 era when higher cumulative doses of CYC, either orally or intravenously, were used and sustained amenorrhoea was used to define ovarian failure [5–12]. With the use of CYC through intravenous route at lower doses for a shorter duration, and development of better markers of ovarian reserve, the incidence of ovarian toxicity with CYC needs reassessment. There is limited data on gonadal toxicity with CYC use in the current era where modified National Institute of Health (NIH) regimens (employing azathioprine or MMF instead of quarterly CYC pulses for maintenance after the initial 6 monthly pulses) or low-dose Euro-Lupus regimen are used [13, 14]. Additionally, the possible negative impact of underlying SLE on ovarian function is an important confounding factor that needs to be taken into consideration [15].

Thus, the present study was done to assess prospectively the ovarian function and reserve in patients of severe lupus treated with CYC, by evaluating changes in menstrual function, hormonal profile [anti-Mullerian hormone (AMH), inhibin B, estradiol, follicle-stimulating hormone (FSH), and luteinizing hormone (LH)], ovarian volume, and antral follicular count with 6 months of CYC therapy (primary objective). We included patients with severe disease treated with MMF (a presumably non-gonadotoxic drug [16]) as an active comparator group (secondary objective), which also allowed us to examine and exclude the possible confounding effect of active disease on ovarian function.

## Methods

This prospective cohort study was conducted at an apex tertiary care centre in North India from July 2015 to November 2016. Adult female SLE subjects in the reproductive age group (18–40 years), satisfying the Systemic Lupus International Collaborating Clinics criteria, and being induced with intravenous CYC or MMF for active disease were included [17]. Exclusion criteria included patients with end-stage renal disease or chronic liver disease; those who had undergone hysterectomy, oophorectomy, or pelvic irradiation; those who were on oral contraceptive pills or hormone replacement therapy; those who had a previous history of polycystic ovarian disease; or those who had inadequate visualisation of ovaries on ultrasonography. We also excluded patients treated with the Euro-Lupus regimen of CYC. Written informed consent was obtained from all subjects.

The CYC group included patients who received induction with monthly intravenous CYC (0.5–0.75 g/m^2^) for 6–9 months followed by azathioprine maintenance, while patients in the MMF group received induction with MMF for 6 months followed by either MMF or azathioprine maintenance. Systemic Lupus Erythematosus Disease Activity Index 2000 (SLEDAI-2K) was used to ascertain disease activity [18]. Serological markers of disease activity, including anti-double-stranded deoxyribonuclease (dsDNA) antibody titres, C3, and C4 levels, were measured. Renal biopsy was performed when indicated, and the International Society of Nephrology/Renal Pathology Society classification was followed.

Estimation of hormone levels in serum and ultrasonographic assessment of ovarian volume and antral follicle count was done at baseline and 6 months after treatment in both the CYC and MMF groups. Fasting venous blood samples for hormone analysis were collected on days 3–5 of the menstrual cycle. In women with amenorrhoea and those with oligomenorrhoea, oral progesterone was given for 1 week, and blood samples were taken on days 1–5 of bleeding or spotting after progesterone withdrawal. Measurement of LH, FSH, estradiol, prolactin, testosterone, and thyroid hormones was performed using an automated analyser (E-2010, Roche Diagnostics, Germany) through electrochemiluminescence immunoassay. AMH and inhibin B levels were measured by enzyme-linked immunosorbent assay (Ansh Labs and Qayee-Bio, respectively). Transvaginal or transabdominal sonography using a 4.5–7.2-MHz (Philips 1422, New Delhi, India) probe was performed on the same day by an experienced gynaecologist (PB) blinded to all patient information. The total number of antral follicles measuring 2–10 mm in diameter and ovarian volumes were assessed. Ovarian volume was calculated as per the ellipsoid formula (0.526 × length × height × width in centimetre), corresponding to the mean volume in both ovaries. Patients who were found to be amenorrhoeic at 6 months were re-evaluated at 1 year. The procedures performed were as per the Declaration of Helsinki, and the study was approved by the institutional ethics committee.

### Statistical analysis

Statistical analysis was performed using Statistical Package for Social Sciences [SPSS Inc., Chicago, IL, version 22]. The normality of quantitative data was assessed using the Kolmogorov-Smirnov test. After confirmation of normality, Student’s *t* test was applied for the comparison of continuous data. The Mann-Whitney *U* test was used for skewed continuous variables. Proportions were compared using the chi-square test or Fisher’s exact test, as appropriate. For dependent samples, a paired *t* test was used for normally distributed data, while the Wilcoxon signed-rank test was used for skewed data. Correlation between groups was assessed using Pearson’s correlation coefficient. A *p* value of < 0.05 was considered statistically significant.

## Results

### Demographic, clinical, and laboratory characteristics

Fifty female SLE patients were included, 25 each in the CYC and MMF groups. The mean (SD) age of study subjects at diagnosis and at the time of recruitment, marital and family status, obstetric and menstrual history, and serological markers of SLE disease activity have been summarised in Table 1.

**Table 1:** Baseline characteristics of patients in the cyclophosphamide (CYC) and mycophenolate mofetil (MMF) groups

Parameter*	CYC (***n*** = 25)	MMF (***n*** = 25)
Age at recruitment (years)	31.4 ± 6.4	28.4 ± 4.4
Age at diagnosis (years)	28.4 ± 6.1	24.1 ± 4.4
Duration of disease from diagnosis (years)	3 ± 2.9	4.3 ± 4
SLEDAI	7.2 ± 2.5	5.8 ± 3.4
*Marital status*		
Unmarried	6 (24%)	8 (32%)
Completed family	13 (52%)	6 (24%)
*Gestational history*		
Median number of successful pregnancies	1	1
Median number of abortions	0	0
*Menstrual cycles at recruitment*		
Regular	24 (96%)	24 (96%)
Oligomenorrhoea	1 (4%)	1 (4%)
ANA positivity	25 (100%)	25 (100%)
dsDNA positivity	21 (84%)	23 (92%)
Hypocomplementaemia	17 (68%)	13 (52%)
Creatinine (mg/dl)	1.38 ± 0.92	0.93 ± 0.56
TSH (mIU/l)	3.37 ± 1.95	2.69 ± 2.24
Testosterone (nmol/l)	0.29 ± 0.35	0.47 ± 0.71
Prolactin (ng/ml)	18.26 ± 7.60	23.31 ± 21.02
Kidney biopsy class		
Class II	1 (5%)	1 (4.5%)
Class III	2 (10%)	5 (23.7%)
Class IV	12 (60%)	6 (27.3%)
Class III + V	0	1 (4.5%)
Class IV + V	5 (25%)	2 (9%)
Class V	0	7 (32%)

*CYC* cyclophosphamide, *MMF* mycophenolate mofetil, *SLEDAI* Systemic Lupus Erythematosus Disease Activity Index, *ANA* antinuclear antibodies, *dsDNA* anti-double-stranded deoxyribonucleic acid antibodies, *TSH* thyroid-stimulating hormone

*Reported as mean ± SD or *n* (%)

The mean (SD) SLEDAI of patients in the CYC group was 7.2 (2.5). All patients had active lupus nephritis at the time of initiation of CYC. Six of these 25 CYC patients had lupus nephritis alone; eight had a concurrent active haematological disease, and six each had concomitant cardiac and neurological involvement along with nephritis, while one had all three systems involved. Renal biopsy was performed in 20 of these patients (remaining three had thrombocytopenia, and two did not consent for biopsy), and the predominant lesion was class IV lupus nephritis [12 (60%) patients], followed by a combination of class IV and V [5 (25%) patients]. The mean (SD) SLEDAI of patients in the MMF group was 5.8 (3.4). All patients had active lupus nephritis at the time of initiation of MMF. Thirteen of these had lupus nephritis alone, eleven had a concurrent active haematological disease, and eight had neurological involvement in addition to nephritis. Renal biopsy was performed in 22 of these patients (remaining three had thrombocytopenia) with the predominant lesion being class V lupus nephritis [7 (32%) patients], followed by class IV [6 (27.3%) patients] and class III [5 (23.7%) patients]. Mean (SD) serum creatinine at baseline was 1.4 (0.9) mg/dl in the CYC group and 0.9 (0.6) mg/dl in the MMF group (*p* = 0.045). The mean cumulative dose of intravenous CYC used was 4.6 ± 1.8 g, while the mean daily dose of MMF was 1280 ± 402 mg. All patients received oral steroids and hydroxychloroquine concomitantly.

### Menstrual disturbances

One subject each in the CYC and MMF groups had oligomenorrhoea at baseline, while all others had regular menstrual cycles before initiation of therapy. During 6 months of follow-up, 14 (56%) subjects receiving CYC developed menstrual irregularities compared to 7 (28%) receiving MMF (*p* = 0.045). Out of the 14 subjects who developed menstrual disturbances with CYC, 6 (42.9%) had oligomenorrhoea, 2 (14.2%) had menorrhagia, and 6 (42.9%) subjects became amenorrhoeic—three of these recovered menses within 3 months of initiation of therapy, two recovered them by 12 months, and only one (4%) subject aged 40 years at recruitment and 41 years at last follow-up had sustained amenorrhoea lasting beyond 12 months. Out of the seven subjects who developed menstrual disturbances with MMF, three (42.86%) had oligomenorrhoea, 2 (28.57%) had menorrhagia, and 2 (28.57%) had amenorrhoea—both of these regained menses within 2 months of initiation of therapy.

### Hormonal characteristics

At baseline, both the CYC and MMF groups had similar levels of serum LH, FSH, estradiol, AMH, and inhibin B (Table 2). After 6 months of therapy with CYC, there was a significant change in levels of FSH, AMH, and inhibin B. While the levels of FSH significantly increased, the levels of AMH and inhibin B decreased significantly (Table 2). There was a non-significant increase in the levels of LH and a non-significant decrease in the levels of estradiol (Table 2). In the MMF group, after 6 months, inhibin B levels showed an increase in the mean levels while the rest of the hormones (LH, FSH, estradiol, and AMH) did not show any significant change. Compared to MMF, the CYC group had significantly lower levels of estradiol, AMH, and inhibin B after 6 months of therapy. Table 2 shows the baseline and 6-month follow-up levels of various hormones tested in both groups. Serum levels of thyroid hormones, prolactin, and testosterone at baseline have been summarised in Table 1.

**Table 2:** Markers of ovarian reserve (serum hormones, ovarian volume, and antral follicular count) at baseline and after 6 months of therapy in the cyclophosphamide (CYC) and mycophenolate mofetil (MMF) groups

Parameter*	CYC (***n*** = 25)			MMF (***n*** = 25)			Between-group ***p*** value (baseline)	Between-group ***p*** value (6 months)
	Baseline	6 months	***p*** value	Baseline	6 months	***p*** value		
LH (IU/ml)	5.8 ± 3.6	17.6 ± 38.4	0.13	9.4 ± 7.7	8.3 ± 5.6	0.55	0.065	0.55
FSH (IU/ml)	5.2 ± 3.4	25.1 ± 46.0	0.03	4.8 ± 1.8	6.1 ± 3.1	0.07	0.938	0.36
E2 (pg/ml)	80.2 ± 57.6	56.1 ± 52.3	0.13	89.6 ± 67.3	113.0 ± 73.3	0.25	0.749	< 0.001
AMH (ng/ml)	5.7 ± 6.7	1.2 ± 1.7	0.002	6.3 ± 3.9	5.8 ± 5.6	0.71	0.061	< 0.001
Inhibin B (pg/ml)	56.1 ± 21.9	16.5 ± 18.0	< 0.001	53.1 ± 24.7	82.9 ± 37.7	0.002	0.547	< 0.001
Ovarian volume (cm^3^)	29.3 ± 15.8	17.9 ± 13.7	0.005	25.7 ± 20.4	26.1 ± 11.1	0.92	0.49	0.02
Antral follicular count	6.4 ± 2.4	5.5 ± 4.2	0.32	6.7 ± 2.5	8.0 ± 3.0	0.07	0.74	0.02

*LH* luteinizing hormone, *FSH* follicle-stimulating hormone, *E2* estradiol, *AMH* anti-Mullerian hormone, *CYC* cyclophosphamide, *MMF* mycophenolate mofetil *Reported as mean ± SD

### Ultrasonographic characteristics

The baseline and end-of-treatment ovarian volume and antral follicular count in the two groups have been summarised in Table 2. The ovarian volume at baseline in the CYC and MMF groups was similar (*p* = 0.49). With 6 months of therapy, the ovarian volume reduced significantly in the CYC group (*p* = 0.005) but remained unchanged in the MMF group (*p* = 0.92) (Table 2). The ovarian volume after 6 months of therapy was significantly lower in the CYC group compared to MMF (*p* = 0.02). The mean antral follicular count at baseline was 6.4 and 6.7 in the CYC and MMF groups, respectively (*p* = 0.74). After 6 months of therapy, it reduced non-significantly (*p* = 0.32) to 5.5 in the CYC group and increased non-significantly to 8 in the MMF group (Table 2). The antral follicular count after 6 months of therapy was significantly lower in the CYC group compared to MMF (*p* = 0.02). Ovarian volume and antral follicle count correlated well with each other in both the groups (*r* = 0.43, *p* = 0.002).

## Discussion

In this prospective cohort study, we assessed the effect of intravenous CYC and oral MMF on ovarian function and reserve in young female patients with severe lupus, through a comprehensive assessment of menstrual irregularities, changes in hormonal profile, and ultrasonographic ovarian parameters with 6 months of therapy. We observed that after 6 months of therapy, three subjects had amenorrhoea in the CYC group compared to none in the MMF group. However, when these patients were followed up to 12 months, two of these recovered menses while only one patient developed sustained amenorrhoea (> 12 months). She was 40 years old at recruitment and 41 years old at last follow-up and had high FSH and LH values with low AMH and inhibin B, suggesting menopause. No premature ovarian failure was noted at 1 year.

However, a subclinical adverse effect on the ovarian reserve was observed with CYC. FSH, AMH, and inhibin B are accepted markers of ovarian reserve, and therapy with CYC led to a significant increase in serum FSH levels along with a significant reduction in AMH and inhibin B levels [19, 20]. AMH is the preferred serological marker for ovarian reserve owing to the constancy of its levels throughout the menstrual cycle, absence of feedback regulations, greater sensitivity to pick up early ovarian dysfunction, and better correlation with primordial follicular pool [20]. However, it still has a poor correlation with future successful pregnancies [21]. Direct visualisation of ovarian volume and antral follicular count using ultrasonography seems to be the best method available currently. In the present study, ovarian volume and antral follicular count were found to reduce with 6 months of therapy with CYC, although the latter did not reach statistical significance. Effect of CYC on FSH, LH, and estradiol has been noted in few studies described previously [6, 7, 9]; however, studies on AMH and follicular counts are limited [16, 21–24]. Effect of CYC on ovarian volumes in SLE has not been assessed prior to this study, although one study did find lower ovarian volumes in patients of SLE not previously exposed to CYC [22]. AMH levels were found to be lower in SLE patients previously exposed to CYC, but doses as per the Euro-Lupus regimen were not found to affect AMH levels [16, 21, 23, 24]. Ma et al. found lower antral follicular counts in patients of SLE, but no difference was found in those with and without CYC therapy [23]. In the absence of long-term follow-up data, the consequences of reduced ovarian reserve on subsequent fertility are difficult to predict. It would be interesting to see whether ovarian volumes remain persistently low leading to impaired fertility, or recover after stopping CYC with a return to normal fertility.

Much work has been done in the field of CYC gonadotoxicity. Risk factors for the development of ovarian failure after CYC therapy include older age, a higher cumulative dose of CYC, and longer treatment duration [9, 25, 26]. The unacceptable rates of ovarian failure with oral CYC were one of the factors which paved the way for a transition from oral to intravenous CYC in clinical practice. Austin et al. had noted ovarian failure in 71% of patients receiving oral CYC compared to 45% of patients receiving intravenous CYC [8]. Boumpas et al. had observed sustained amenorrhoea in 12% patients on a short course of intravenous CYC (0.5–1 g/m^2^ for 7 pulses) compared to 39% on a long course (more than 15 pulses) [11]. They also observed that amenorrhoea developed in 62% of women over 30 years of age compared to 12% in those less than 25 years. Similar differences were noted by Mok et al., who observed amenorrhoea in 50% of women aged over 40 years compared to 14% in those less than 30 years [9]. Logistic regression analysis confirmed these parameters as independent risk factors of CYC-induced ovarian failure [26]. However, studies reporting high rates of ovarian failure (as high as 59%) with CYC belonged chiefly to the era of oral CYC and NIH regimen of intravenous CYC. Subsequently, the lower dose St. Thomas or Euro-Lupus regimen and modifications of the NIH regimen (where quarterly pulses of CYC after the initial 6 monthly pulses have been replaced by azathioprine or MMF) have been developed and have become standard of care. Indeed, the Euro-Lupus nephritis trial found the rates of sustained amenorrhoea in the low-dose and high-dose arms to be 4.5% and 4.4%, respectively [13]. However, no premature menopause was noted in the low-dose arm, whereas the single patient who developed premature menopause in the high-dose arm had required the use of oral CYC for severe disease [13]. There is a paucity of data on the ovarian toxicity of modified NIH regimens of CYC. A 6-month randomised controlled trial from the Indian subcontinent compared modified NIH and Euro-Lupus regimens in lupus nephritis and found the rates of sustained amenorrhoea to be 5% and 2% in the two arms, respectively [14]. In our study, the rate of sustained (> 12 months) amenorrhoea with CYC was 4% but no premature ovarian failure was observed at a mean cumulative dose of 4.6 ± 1.8 g.

It is also important to note that the use of sustained amenorrhoea as a surrogate for ovarian failure (as employed in most of the studies mentioned above) can lead to erroneous estimates of ovarian dysfunction. A strategy employing measurement of hormone levels and direct sonographic visualisation of ovaries for ascertaining ovarian volume and antral follicular counts, as done in the current study, is advisable for more reliable estimates and detection of subclinical ovarian damage.

SLE, by itself, has been found to have a negative effect on ovarian reserve in premenopausal women in many studies [22, 23], although a few others have reported conflicting results [27]. The mechanism is not entirely known but is presumed to be multifactorial [15]. Autoimmune oophoritis and the effect of chronic inflammation on the hypothalamic-pituitary-ovarian axis are variable contributory factors [15]. To address this critical confounding factor, we included a comparator group comprising of SLE patients with active disease treated with MMF (a drug with little or no gonadotoxicity), but did not find any change in hormonal or sonographic markers of ovarian reserve.

Gonadotropin-releasing hormone (GnRH) analogs, by creation of an artificial prepubertal hypogonadotropic milieu and reduced utero-ovarian perfusion, may have a role in the prevention of chemotherapy-induced premature ovarian failure in an oncological setting [28]. However, current evidence of their efficacy in autoimmune rheumatic diseases, where much lower doses of gonadotoxic drugs are used, is limited to a few small non-randomised studies. Also, its use comes at the cost of undesirable side effects and a possibility of worsening of background lupus [29]. It is possible that the subclinical effect of CYC on ovarian function and reserve seen in our study and previous studies could have been further circumvented by the use of GnRH analogs in susceptible women.

The limitations of the current study include its observational design, with no randomisation for choice of treatment (CYC versus MMF), predisposing to a potential selection bias. A relatively small number of subjects were included, and a follow-up duration of 1 year precluded the assessment of subsequent fertility. The increase in inhibin B levels noted with MMF could not be completely accounted for. The findings of the present study are not applicable to premenopausal, fertile women above 40 years of age.

The merits of the study include a robust prospective study design with inclusion, for the first time, of patients with severe disease treated with MMF as a comparator to examine and exclude the possible confounding effect of SLE disease activity on gonadal function. Ovarian function was assessed holistically through a detailed clinical assessment of menstrual irregularities; biochemical assessment of hormonal markers including FSH, LH, estradiol, AMH, and inhibin B; and sonographic assessment of ovarian volume and antral follicular count.

## Conclusions

The adverse effects of induction with moderate-dose, monthly (0.5–0.75 g/m^2^ for 6–9 months) intravenous CYC (modified NIH regimen) on the ovarian function were predominantly subclinical, with a negative effect on ovarian reserve. The majority of patients who suffered from amenorrhoea recovered menses subsequently, leading to a low rate of sustained amenorrhoea at 1 year (4%). No premature ovarian failure was noted at 1 year. No adverse effect on the ovarian reserve was seen in patients treated with MMF, despite having severe lupus disease activity. The use of currently employed regimens of intravenous CYC for induction therapy may thus not be restricted in young SLE females with incomplete families for fear of gonadotoxicity, especially in severe life- or organ-threatening cases, where CYC is superior to other drugs and the benefits outweigh this subclinical risk.

## Significance

### What is already known in literature?

- Cyclophosphamide is the preferred first-line agent for the management of many severe manifestations of lupus. However, it is notorious for causing premature ovarian failure, which has far-reaching consequences. Despite modifications of the original NIH regimen and the use of lower dose Euro-lupus regimens, concerns about its gonadotoxicity still remain widely prevalent and often preclude its use in young unmarried women or those with incomplete families. Lupus per se is also believed to affect ovarian function negatively.

### What are the major methodological merits of the study?

- We used severe lupus patients treated with mycophenolate (a non-gonadotoxic drug) as a comparator group to delineate the confounding effect of lupus disease activity on ovarian function and reserve. We used a robust combination of objective sonographic and hormonal parameters to study ovarian toxicity.

### What does this study add to the existing literature?

- Ovarian dysfunction with intravenous cyclophosphamide (0.5–0.75 g/m^2^ monthly for 6–9 months; modified NIH regimen being used in many centres globally) is mild and predominantly subclinical. Although negative effects on ovarian reserve were observed, no premature ovarian failure was noted.
- Lupus disease activity did not have a bearing on ovarian function or reserve.

### How do the results of this study impact clinical practice?

Use of intravenous cyclophosphamide may not be restricted (at least for induction) in young lupus patients for fear of gonadotoxicity, especially in severe life- or organ-threatening disease, where the benefits outweigh the subclinical risk.

## Data Availability

All data generated or analysed during this study are included in this published article.

## Abbreviations

AMH: Anti-Mullerian hormone
CYC: Cyclophosphamide
dsDNA: Double-stranded deoxyribonuclease
FSH: Follicle-stimulating hormone
GnRH: Gonadotropin-releasing hormone
LH: Luteinizing hormone
MMF: Mycophenolate mofetil
NIH: National Institute of Health
SLE: Systemic lupus erythematosus
SLEDAI: Systemic Lupus Erythematosus Disease Activity Index
